# A predictive model to identify optimal candidates for surgery among patients with metastatic colorectal cancer

**DOI:** 10.3389/fonc.2025.1573431

**Published:** 2025-06-05

**Authors:** Xiqiang Zhang, Zhaoyi Jing, Longchao Wu, Ze Tao, Dandan Lu

**Affiliations:** ^1^ The First Clinical College, Shandong University, Jinan, Shandong, China; ^2^ The First Clinical College, Shandong University of Traditional Chinese Medicine, Jinan, China; ^3^ Day Surgery Ward, Qilu Hospital of Shandong University, Jinan, China

**Keywords:** primary tumor resection, machine learning, metastatic colorectal cancer, cancer specific survival, predictive model

## Abstract

**Purpose:**

To improve clinical decision-making, we developed a predictive model to identify metastatic colorectal cancer (mCRC) patients who might benefit from primary tumor resection (PTR).

**Patients and Methods:**

We extracted clinical data of stage IV CRC patients between 2010 and 2019 from the Surveillance, Epidemiology, and End Results database. Propensity score matching (PSM) was used to balance confounding factors by categorizing patients into surgery and non-surgery groups. To identify independent predictors of cancer-specific survival (CSS), we used multivariate Cox regression analysis. We further sorted patients who underwent surgery into benefit and non-benefit groups based on the median CSS of the non-surgery group; subsequently, we split the groups into training and test sets at a ratio of 6:4. To construct predictive models, we used the Boruta selection method to further filter variables, focusing on whether patients benefited from the surgery, based on key predictive factors.

**Results:**

We identified 23,649 mCRC patients, of whom 80.97% (19,148) underwent PTR. After PSM, compared to no surgical intervention, surgical intervention was independently associated with an extended median CSS [median: 22 *vs*. 12 months; HR: 2.323, P < 0.001]. Among the nine machine learning models, the Categorical Boosting model performed the best but was still slightly inferior to traditional logistic regression. The traditional logistic regression model showed good discriminative ability in both the training (area under the curve [AUC]: 0.727 [0.699-0.756]) and test (AUC: 0.741 [0.706-0.776]) sets.

**Conclusion:**

We achieved a predictive model which could identify optimal candidates for PTR among mCRC patients with high accuracy.

## 1 Introduction

Globally, colorectal cancer (CRC) ranks third in the frequency of digestive system malignancies (1). In approximately 20% of cases, distant metastases are present at the time of initial diagnosis (2, 3), resulting in a 5-year survival rate of <14%, which is further reduced in rectal cancer patients. Current treatments for metastatic CRC (mCRC) focus on improving both cancer-specific survival (CSS) and overall survival (OS), leading to the widespread utilization of systemic and palliative interventions (4). The National Comprehensive Cancer Network guidelines suggest primary tumor resection (PTR) as a surgical option for stage IV CRC patients (5). PTR, with chemotherapy, improves OS and CSS in certain patient populations (6). One study demonstrated a median OS of 18.3 months in patients that received PTR with chemotherapy, compared to 8.4 months for those who underwent chemotherapy alone (7). Despite notable advancements in the efficacy of chemotherapies, approximately 70% of patients choose PTR (8, 9). Nevertheless, determining treatment strategies for unresectable stage IV CRC depends on the expertise, discernment, and individual preferences of the attending clinician. Our knowledge about which patients derive the greatest benefit from surgery remains incomplete. Hence, a predictive model of effectively recognizing potential candidates for PTR is urgently needed.

Recently, notable advancements in machine learning algorithms in medicine have been made, emphasizing oncological applications (10). Nevertheless, the efficacies of these machine-learning models depend on the availability of substantial datasets for training, which is challenging in certain medical settings. Additionally, the opaque nature of these models can impede the comprehension of decision-making mechanisms by medical practitioners and patients. Therefore, using machine learning to address specific clinical issues is not always optimal. Traditional logistic regression models remain viable alternatives (11), providing clear and interpretable visual representations that enhance comprehensibility regarding outcomes (12, 13).

Herein, we constructed a predictive model employing data from SEER database to identify candidates suitable for PTR among mCRC patients. Additionally, the predictive performance of traditional logistic regression and machine learning models are compared.

## 2 Methods

### 2.1 Research participants

We extracted data from CRC patients between 2004 and 2019 using the SEER*Stat software (version 8.4.2), with tumor site codes ranging from C18.0, C18.2-C18.9, C19.9, and C20.9. Patients with histologically confirmed CRC according to the 7th edition of AJCC TNM classification were included. The exclusion criteria were more than one primary tumor and missing or incomplete data regarding grade, TNM stage, treatment information, or survival time.

### 2.2 Balancing the dataset and feature selection

The patients were classified into surgery and non-surgery cohorts depending on the conduct of PTR. To balance baseline characteristics between the two groups, propensity score matching (PSM) was performed using a logistic regression model and a 1:1 matching ratio with a caliper value of 0.01, based on the nearest neighbor matching method.

Cancer-specific survival (CSS), overall survival (OS) and survival time were retrieved from the Surveillance, Epidemiology, and End Results (SEER) database. In this study, the median follow-up duration was 65 months, indicating that the study population had a relatively adequate overall follow-up period. The starting point for calculating survival time was defined as the date of diagnosis of mCRC. CSS was defined as the time from diagnosis to cancer-related death, and OS as the time from diagnosis to all-causes death. Kaplan-Meier analysis was used to estimate the median survival times and 95% confidence intervals (CIs); log-rank tests were used to compare variations in CSS and OS between the groups. Multivariate Cox proportional hazards regression identified independent prognostic factors for CSS, with statistical significance set at P<0.05. The study employed the Fine-Gray sub distribution hazard model to perform competing risk analysis, aiming to accurately evaluate the impact of PTR on the risk of colorectal cancer–specific mortality. In this model, cancer-specific death was defined as the primary event, while non-cancer-related death—including deaths potentially associated with surgical complications—was treated as a competing event. Cumulative incidence functions (CIFs) were calculated and plotted to visualize the time-dependent probability of event occurrence across different groups. Based on the hypothesis that patients who benefit from PTR have an extended median CSS than those without surgery, patients who underwent surgery were further categorized into benefit and non-benefit groups. Additionally, patients were split into training and test sets in a 6:4 ratio (random seed number 125) in the surgery group; factors independently affecting CSS and available preoperatively in the multivariate Cox analysis were further identified using the Boruta method (100 iterations) (14).

### 2.3 Model building and evaluation

The features selected in the above steps were introduced sequentially into nine machine learning algorithms—Naive Bayes, Light Gradient Boosting Machine, Gradient Boosting Trees, Support Vector Machine, Adaptive Boosting, Support Vector Machine, Categorical Boosting (CatBoost) model, logistic regression, eXtreme Gradient Boosting, Random Forest—and a traditional logistic regression model. Model and hyperparameter optimizations were performed on the training set, with the test set used for performance comparison to avoid overfitting; bootstrapping methods were used for internal validation. The model’s overall evaluation metrics included the area under the curve (AUC) (15, 16), accuracy, precision, recall, and F1 score; through calibration and decision curves, the model fit and clinical utility were compared.

Regarding the traditional logistic model, initial validation was followed by risk score calculation using the MedCalc software (version 22.1.0.0). The Jouden index was used to identify the optimal risk score threshold, allowing risk stratification for all subjects. Kaplan-Meier survival analysis was performed to evaluate any significant differences in prognosis between different risk groups.

### 2.4 Feature importance and model interpretability analysis

Model evaluation metrics were analyzed to identify the best-performing machine-learning model. Feature importance analysis was performed to identify and quantify the influence of each feature on prediction outcomes. Shapley Additive Explanations were employed to rigorously evaluate the predictive effectiveness of the models (17).

### 2.5 Statistical analysis

As the continuous variables did not follow a normal distribution, intergroup comparisons were conducted using rank-sum tests, and the results were presented as medians and interquartile ranges. Categorical variables were examined with the χ2 test (Fisher’s exact test was applied when anticipated counts were <5). All statistical analyses were performed using R version 4.2.1.

## 3 Results

### 3.1 Baseline characteristics before and after PSM

Of the 608,951 CRC cases between 2004 and 2019 in the SEER database, 23,649 stage IV cases met the inclusion criteria (**Supplementary Figure S1**); Among these, 19,148 (80.97%) underwent PTR. After PSM, 2,558 pairs were included in the survival analysis, achieving a statistical balance across all baseline characteristics (P>0.05) (**Table 1**). Before PSM, many variables had standardized mean differences exceeding the traditional threshold of 0.1; PSM effectively reduced potential selection bias (**Supplementary Figure S2A**). The details of the matched variables are shown in **Supplementary Figure S2B**.

**Table 1:** Characteristics for study population by study groups before and after PSM.

Variable names	Before PSM			After PSM		
	Surgery to primary site (n=19148),n%	Non-surgery to primary site (n=4501), n%	P value	Surgery to primary site (n=2558), n%	Non-surgery to primary site (n=2558), n%	P value
Age	62 (52-72)	62 (53-72)	0.03	62 (53-71)	61 (52-72)	0.54
Gender						
Male	9916 (51.79)	2660 (59.10)	<0.01	1475 (57.66)	1495 (58.44)	0.59
Female	9232 (48.21)	1841 (40.90)		1083 (42.34)	1063 (41.56)	
Marriage						
Married	10152 (53.02)	2216 (49.23)	<0.01	1325 (51.80)	1325 (51.80)	1
Never married/other	8996 (46.98)	2285 (50.77)		1233 (48.20)	1233 (48.20)	
Race						
White	14534 (75.90)	3433 (76.27)	0.8	1955 (76.43)	1949 (76.19)	0.97
Black	2602 (13.59)	610 (13.55)		335 (13.10)	341 (13.33)	
Other	2012 (10.51)	458 (10.18)		268 (10.48)	268 (10.48)	
Site						
Right-hemicolon	9295 (48.54)	1115 (24.77)	<0.01	648 (25.33)	684 (26.74)	0.43
Left-hemicolon	6209 (32.43)	928 (20.62)		573 (22.40)	579 (22.63)	
Rectum	3644 (19.03)	2458 (54.61)		1337 (52.27)	1295 (50.63)	
Histologic						
Adenomas and adenocarcinomas	16446 (85.89)	4127 (91.69)	<0.01	2346 (91.71)	2335 (91.28)	0.64
Cystic, mucinous and serous neoplasms	2449 (12.79)	206 (4.58)		159 (6.22)	160 (6.25)	
Other	253 (1.32)	168 (3.73)		53 (2.07)	63 (2.46)	
Grade						
I	1127 (5.89)	319 (7.09)	<0.01	180 (7.04)	180 (7.04)	0.42
II	12365 (64.58)	3069 (68.18)		1802 (70.45)	1754 (68.57)	
III	4593 (23.99)	1021 (22.68)		521 (20.37)	559 (21.85)	
IV	1063 (5.55)	92 (2.04)		55 (2.15)	65 (2.54)	
T						
T1	326 (1.70)	1653 (36.73)	<0.01	292 (11.42)	332 (12.98)	0.44
T2	508 (2.65)	170 (3.78)		118 (4.61)	116 (4.53)	
T3	9744 (50.89)	1483 (32.95)		1267 (49.53)	1228 (48.01)	
T4a	5215 (27.24)	167 (3.71)		151 (5.90)	163 (6.37)	
T4b	3355 (17.52)	1028 (22.84)		730 (28.54)	719 (28.11)	
N						
N0	3612 (18.86)	2478 (55.05)	<0.01	1129 (44.14)	1126 (44.02)	0.94
N1	6428 (33.57)	1781 (39.57)		1198 (46.83)	1200 (46.91)	
N1c	672 (3.51)	46 (1.02)		48 (1.88)	41 (1.60)	
N2a	3366 (17.58)	86 (1.91)		82 (3.21)	84 (3.28)	
N2b	5070 (26.48)	110 (2.44)		101 (3.95)	107 (4.18)	
M						
M1a	11634 (60.76)	2126 (47.23)	<0.01	1356 (53.01)	1343 (52.50)	0.74
M1b	7514 (39.24)	2375 (52.77)		1202 (46.99)	1215 (47.50)	
Radiation						
Yes	1926 (10.06)	1042 (23.15)	<0.01	645 (25.22)	628 (24.55)	0.6
None/unknown	17222 (89.94)	3459 (76.85)		1913 (74.78)	1930 (75.45)	
Chemotherapy						
Yes	13753 (71.82)	3261 (72.45)	0.41	1904 (74.43)	1894 (74.04)	0.77
None/unknown	5395 (28.18)	1240 (27.55)		654 (25.57)	664 (25.96)	
Surgery other sites						
Yes	5806 (30.32)	257 (5.71)	<0.01	224 (8.76)	236 (9.23)	0.59
None/unknown	13342 (69.68)	4244 (94.29)		2334 (91.24)	2322 (90.77)	

Includes colorectal cancer cases from the SEER database spanning 2004 to 2019, providing the number of cases and column percentages for each variable.

PSM, propensity-score matching.

### 3.2 PTR as an independent predictor of survival in stage IV CRC

Patients undergoing PTR exhibited extended OS and CSS (**Supplementary Figure S3**). The median CSS for the surgery cohort was 22 months (95% CI, 8-41) compared with that of 12 months (95% CI, 4-21) for the non-surgery cohort (**Supplementary Table S1**). In the surgery and non-surgery groups, the 1-year CSS rates were 68.64% and 50.77% respectively, and the 3-year CSS rates were 16.01% and 10.11%, respectively. Multivariable Cox regression further confirmed (**Table 2**) that PTR was independently correlated with better OS (hazard ratio [HR]=2.29, 95% CI, 2.15-2.45, P<0.001) and CSS (HR=2.32, 95% CI, 2.17-2.48, P<0.001). However, in the competing risk analysis (**Supplementary Figure S4**), the CIF curves for cancer-specific mortality were nearly overlapping between the surgery and non-surgery groups, with no statistically significant difference (P=0.633). This indicates that, when accounting for non-cancer-related deaths as competing events, PTR did not significantly improve cancer-specific survival. Additionally, the incidence of non-cancer-related death was slightly higher in the surgery group, although not statistically significant (P=0.925). This may suggest a potential risk of surgery-related complications in a subset of patients. These findings further emphasize that PTR should not be routinely applied to all patients with stage IV disease. Instead, it is essential to identify individuals who are most likely to derive true benefit from surgical intervention. Additionally, chemotherapy, age, tumor location, race, marital status, TNM stage, histology and surgery at distant sites were independent factors affecting survival, whereas sex and radiotherapy had no significant impact.

**Table 2:** Multivariate cox analysis for OS and CSS among PSM population.

Variable names	OS		CSS	
	HR (95%CI)	P value	HR (95%CI)	P value
Age	1.015 (1.012-1.017)	0	1.015 (1.012-1.017)	0
Marriage				
married	Reference		Reference	
never married/other	1.182 (1.109-1.260)	0	1.175 (1.100-1.256)	0
Race				
White	Reference		Reference	
Black	1.129 (1.029-1.239)	0.01	1.140 (1.035-1.256)	0.008
Other	0.926 (0.834-1.028)	0.15	0.947 (0.848-1.057)	0.333
Site				
Right-hemicolon	Reference		Reference	
Left-hemicolon	0.903 (0.825-0.988)	0.027	0.907 (0.826-0.996)	0.041
Rectum	0.811 (0.743-0.884)	0	0.816 (0.746-0.894)	0
Histologic				
Adenomas and adenocarcinomas	Reference		Reference	
Cystic, mucinous and serous neoplasms	0.860 (0.752-0.984)	0.029	0.862 (0.749-0.993)	0.039
Other	1.162 (0.943-1.433)	0.159	1.144 (0.919-1.425)	0.228
Grade				
I	Reference		Reference	
II	1.437 (1.253-1.646)	0	1.485 (1.286-1.715)	0
III	2.191 (1.893-2.537)	0	2.274 (1.949-2.654)	0
IV	2.557 (2.032-3.218)	0	2.657 (2.092-3.375)	0
T				
T1	Reference		Reference	
T2	0.799 (0.670-0.954)	0.013	0.823 (0.684-0.991)	0.040
T3	0.810 (0.729-0.899)	0	0.817 (0.732-0.912)	0
T4a	1.001 (0.856-1.170)	0.994	1.008 (0.857-1.187)	0.922
T4b	0.994 (0.892-1.109)	0.919	1.006 (0.898-1.127)	0.917
N				
N0	Reference		Reference	
N1	1.099 (1.025-1.179)	0.008	1.097 (1.020-1.180)	0.012
N1c	1.194 (0.924-1.541)	0.175	1.197 (0.921-1.555)	0.180
N2a	1.006 (0.838-1.207)	0.948	1.020 (0.847-1.230)	0.833
N2b	1.318 (1.124-1.546)	0.001	1.338 (1.136-1.574)	0
M				
M1a	Reference		Reference	
M1b	1.451 (1.359-1.550)	0	1.484 (1.386-1.589)	0
Radiation				
Yes	Reference		Reference	
None/unknown	1.076 (0.988-1.172)	0.093	1.089 (0.996-1.191)	0.061
Chemotherapy				
Yes	Reference		Reference	
None/unknown	2.658 (2.462-2.870)	0	2.698 (2.491-2.921)	0
Surgery other sites				
Yes	Reference		Reference	
None/unknown	1.290 (1.145-1.453)	0	1.289 (1.138-1.460)	0
Surgery				
Yes	Reference		Reference	
No	2.296 (2.151-2.451)	0	2.323 (2.171-2.486)	0

PSM, propensity score matching; OS, overall survival; CSS, cancer specific survival; HR, hazard ratio.

### 3.3 Variable feature selection

**Figure 1:**
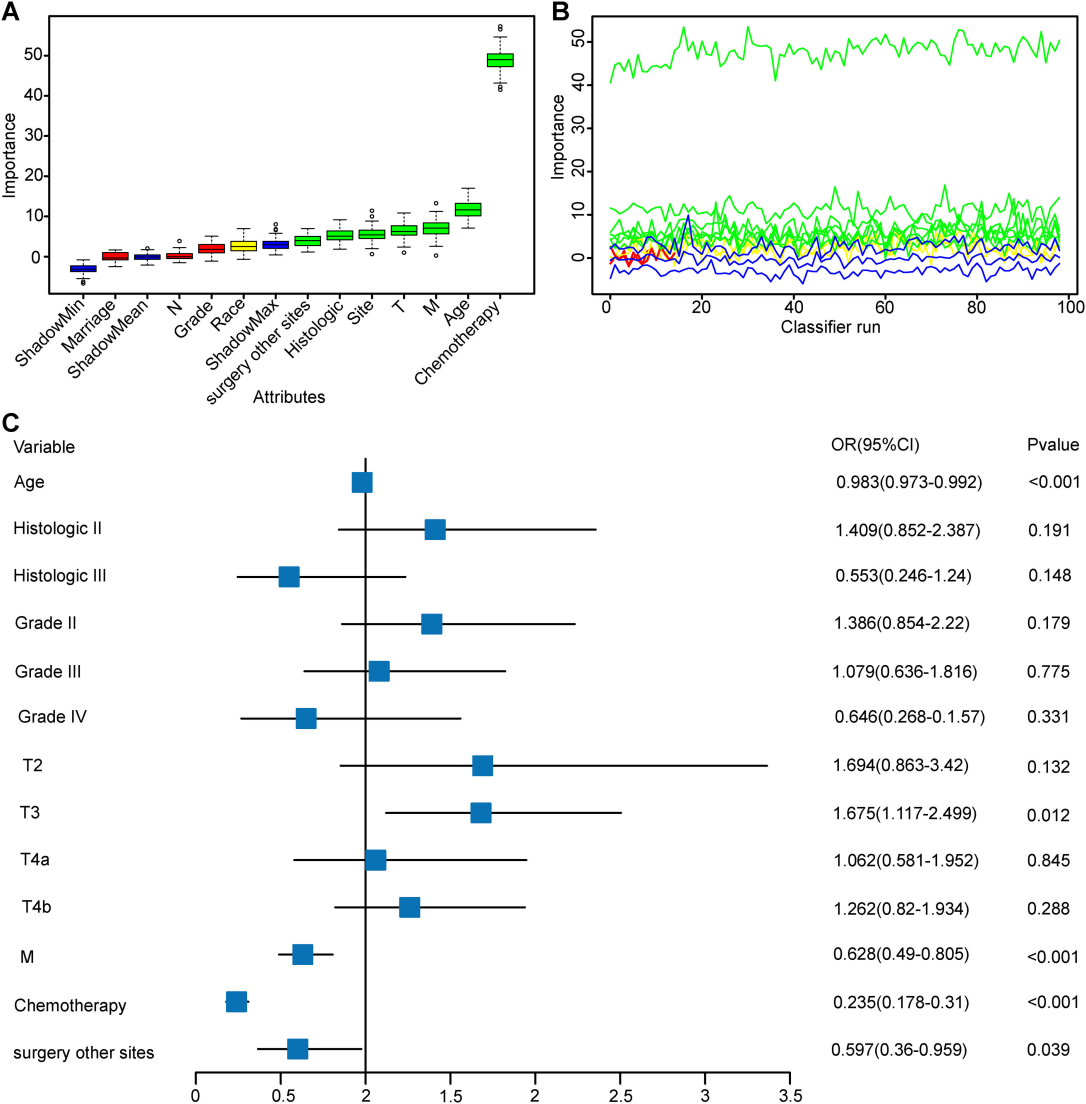
Boruta Feature Selection Method for Variable Importance and Multifactorial Logistic Regression Analysis. This figure comprehensively presents the results of feature selection and multifactorial logistic regression analysis, evaluating the contribution and predictive power of variables within the model. **(A)** Variable Importance Plot: Displays the importance of variables in Boruta feature selection. **(B)** Variable Feature Detail Selection Plot: Illustrates the variability in importance of each variable across 100 classifier runs. **(C)** Multifactorial Logistic Regression Forest Plot: Based on variables selected by the Boruta method, this plot shows the effect size and statistical significance of each variable in a multifactorial logistic regression model. Abbreviation: Histologic II, Cystic, mucinous and serous neoplasms; Histologic III, other.

The surgery group was split into training and test sets to ensure a baseline characteristic balance between the groups (**Supplementary Table S2**). The Boruta method was used to select variables, including age, race, histology, Grade, T stage, M stage, chemotherapy, and surgery at distant sites (**Figures 1A–C**; **Supplementary Table S3**). When excluding race, no significant changes in the AUC values of the training and test sets were observed; thus, the residual variables were s chosen as per the Boruta algorithm. **Figure 1A** shows the variable importance ranking.

### 3.4 Comparison of predictive performance between traditional logistic regression and nine machine learning models

**Figure 2:**
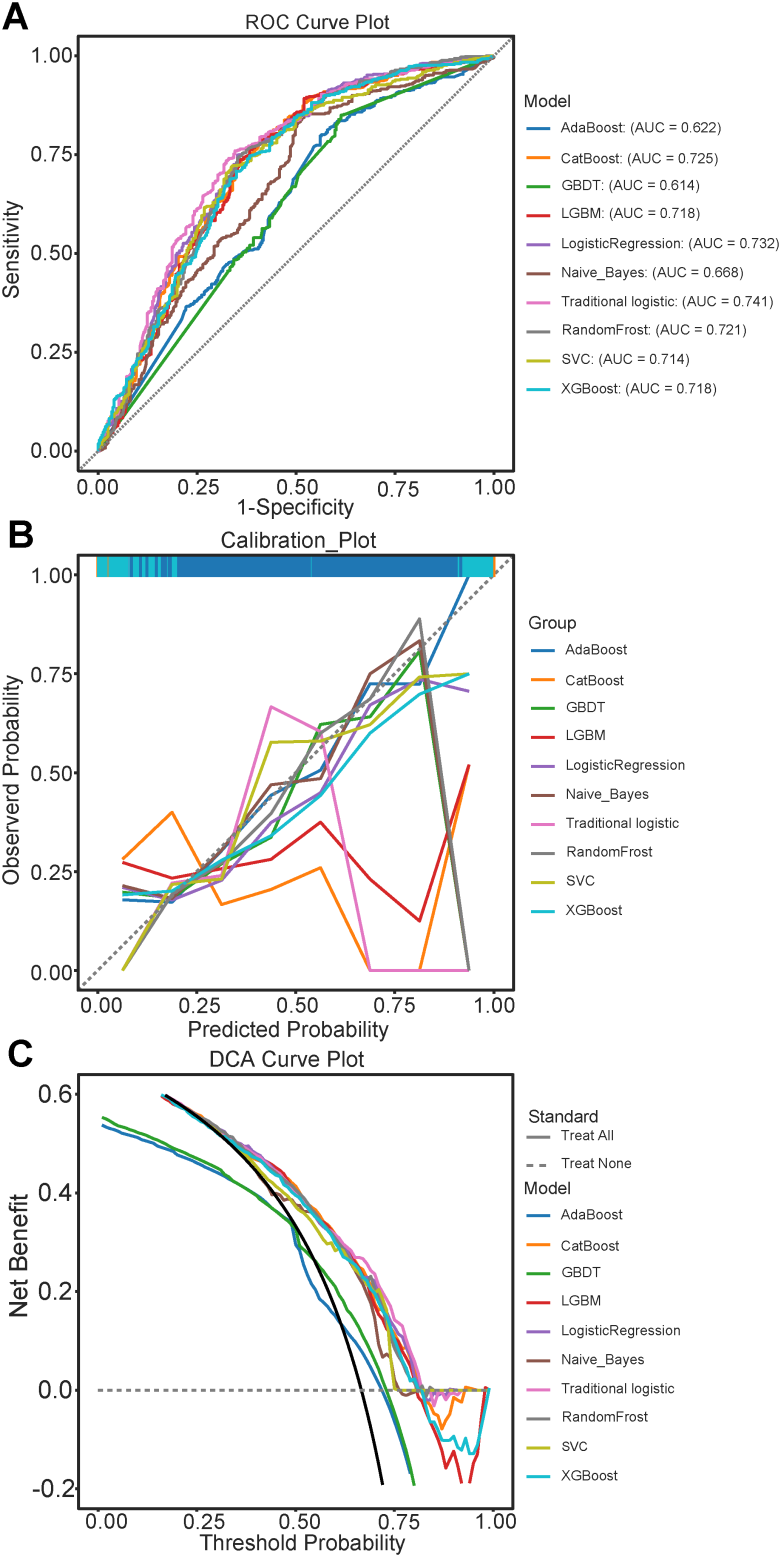
Evaluation of Predictive Performance of models in the test set queue. **(A)** Receiver Operating Characteristic (ROC) curves for various models, illustrating the trade-off between sensitivity and 1-specificity; **(B)** Calibration curves comparing the predicted probabilities and observed outcomes across different models: **(C)** Decision Curve Analysis (DCA) illustrating the net benefit of each model at different threshold probabilities. AdaBoost, Adaptive Boosting; CatBoost, Categorical Boosting; GBDT, Gradient Boosting Trees; LightGBM, Light Gradient Boosting Machine; SVM, Support vector machine; XGBoost, eXtreme Gradient Boosting.

**Supplementary Table S4** presents the predictive accuracies and performances of the nine machine learning algorithms. The CatBoost model achieved the highest accuracy, MC, F1 score, and recall rate of 0.747, 0.398, 0.824, and 0.885, respectively. Traditional logistic regression had better predictive accuracy and performance (**Figure 2A**), reaching AUC values of 0.725 (95% CI: 0.695-0.753) and 0.741 (95% CI: 0.706-0.776) respectively, and exhibited better consistency (**Figure 2B**). Decision curve analysis (DCA) (**Figure 2C**) indicated higher benefits regarding patient decision-making for the traditional logistic regression model.

### 3.5 Evaluation and rational analysis of the predictive nomogram

**Figure 3:**
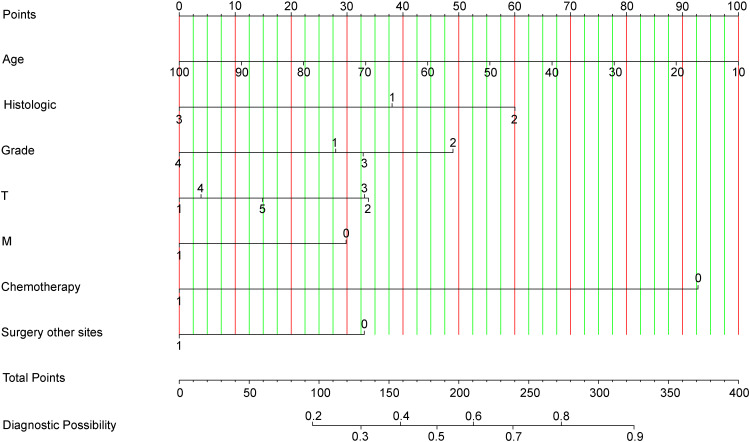
Nomogram for Predicting Optimal Candidates for Primary Tumor Resection. Chemotherapy: ‘Chemotherapy0’ indicates patients who received chemotherapy; ‘Chemotherapy1’ refers to patients whose chemotherapy status is unknown or not administered. Grade: Tumor grades are categorized as Grade1 (Grade I), Grade2 (Grade II), Grade3 (Grade III), and Grade4 (Grade IV). Histologic Type: ‘Histologic1’ represents adenomas and adenocarcinomas; ‘Histologic2’ encompasses cystic, mucinous, and serous neoplasms; ‘Histologic3’ includes other types. Surgery at Other Sites: ‘Surgery other sites0’ denotes patients who received therapy at sites other than the primary tumor location; ‘Surgery other sites1’ indicates status unknown or not administered. Metastasis: ‘M0’ corresponds to M1a; ‘M1’ corresponds to M1b, categorizing the extent of metastatic spread.

By integrating seven diagnosis-related predictive indices, we constructed an optimized nomogram to predict candidates suitable for PTR among stage IV CRC patients (**Figure 3**). The total score for a patient was determined by finding the scores associated with each predictive index for the patient values on the row and summing them to the “Points” row, which was mapped to the “Diagnostic Possibility” line to estimate the patient’s diagnostic probability; the predicted scores are detailed in the **Supplementary File**.

#### 3.5.1 Discrimination

**Figure 4:**
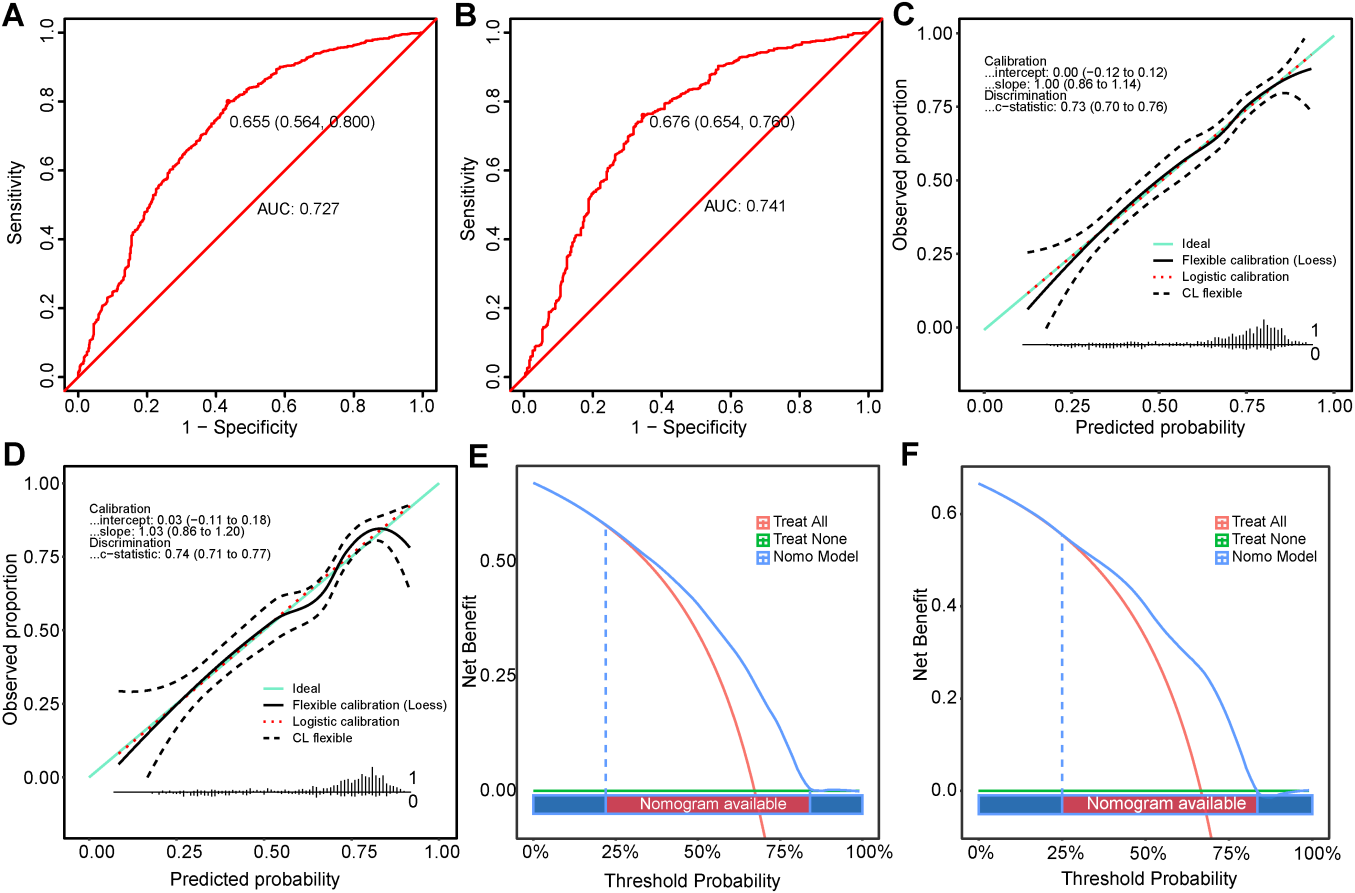
Performance Evaluation of the Nomogram in Predicting Optimal Candidates. **(A, B)** show the ROC curves for the training set and the validation set. **(C, D)** depict the calibration curves for the training and validation sets **(E, F)** illustrate the DCA for the training and validation sets. DCA, decision curve analysis; ROC, receiver operating characteristic.

The receiver operating characteristic (ROC) curves for the training and test sets yielded AUC values of 0.727 (95% CI: 0.699-0.756) and 0.741 (95% CI: 0.706-0.776) respectively, demonstrating consistent predictive capabilities and good performance for unknown data (**Figures 4A, B**). The ROC curves and CIs trained with bootstrapping (500 times) for the training and test sets can be found in **Supplementary Figure S3**.

#### 3.5.2 Calibration

The calibration curves for the training (**Figure 4C**) and test sets (**Figure 4D**) showed a good model fit, which was corroborated by the Hosmer-Lemeshow goodness-of-fit test, with a χ²=5.334, P=0.721 for the training set and χ²=13.861, P=0.085 for the test set.

#### 3.5.3 Clinical utility

DCA for both populations showed that the nomogram had higher benefits within the threshold probability ranges 22%-84% and 25%-83.5% (**Figures 4E, F**) for predicting PTR. When using this predictive model for risk stratification in 1000 patients, the converging trends of the two curves provided an intuitive tool for clinical decision-making, identifying the optimal high-risk threshold at a specific cost-benefit ratio (**Supplementary Figures S5E**, **S5F**).

#### 3.5.4 Rational analysis

In both populations, the AUC values and optimal threshold probabilities based on nomogram predictions were significantly better than those of single variables (**Supplementary Figure S6**). **Supplementary Table S5** presents the statistical analyses of performance metrics for the model. Moreover, the nomogram score for the benefit group exceeded that of the non-benefit group across both the training (**Supplementary Figure S7A**) and test sets (**Supplementary Figure S7B**), showing significant statistical differences. Subsequent logistic regression based on the nomogram score (**Supplementary Figure S7C**) showed increasing odds ratios from the first to the fourth quartiles, all of which were statistically significant (P < 0.001).

### 3.6 Clinical application of the nomogram

**Figure 5:**
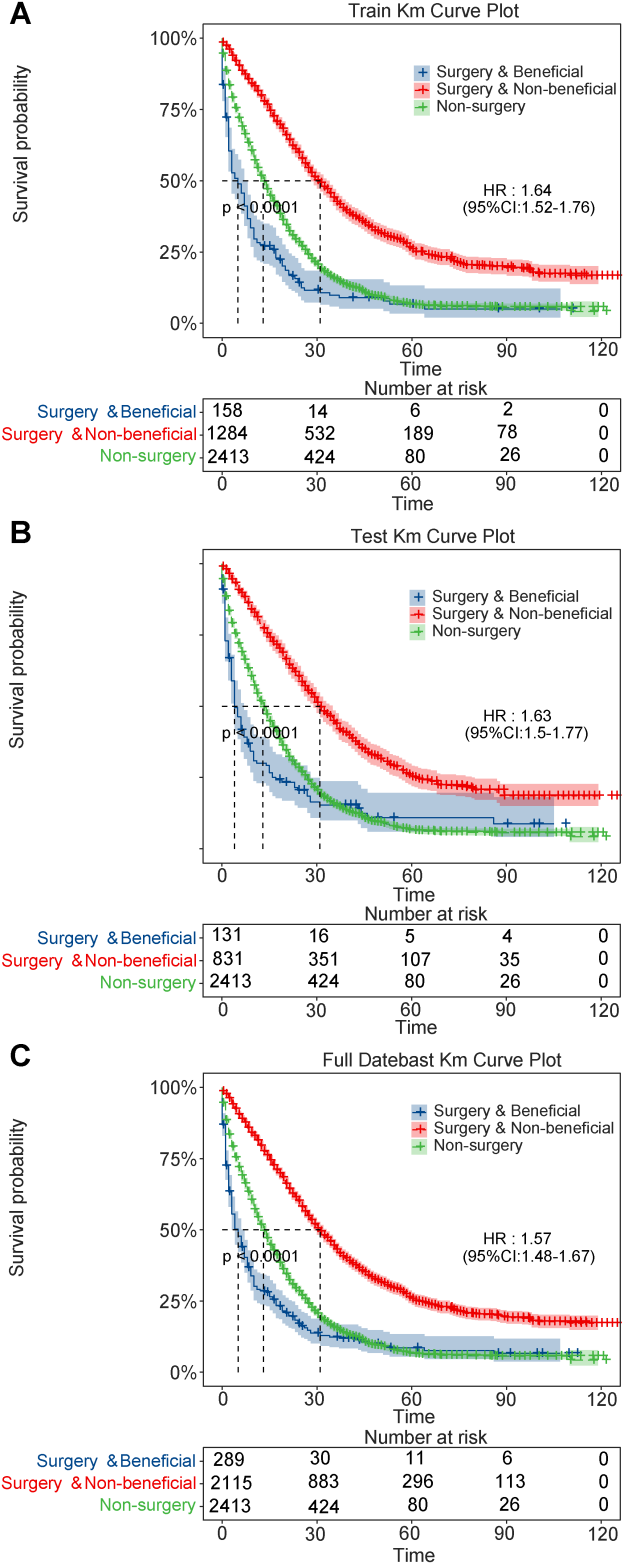
Kaplan-Meier survival curves for patients with metastatic colorectal cancer. **(A)** training set; **(B)** validation set; **(C)** full dataset.

Kaplan-Meier analysis accurately distinguished between the different groups regarding survival prognosis across the training and test sets (**Figure 5**). CSS was markedly higher in the benefit group than in the non-benefit (HR=0.329, 95% CI: 0.268-0.405, P<0.001) and the non-surgery (HR=0.449, 95% CI: 0.408-0.495, P<0.001) groups within the test set. Additionally, the CSS of the non-surgery group was markedly elevated than that of the non-benefit group (HR = 0.733, 95% CI: 0.604-0.889, P=0.002), indicating that the nomogram effectively identified patients who could benefit from PTR. However, some patients may be better suited for personalized nonsurgical treatment or palliative care.

### 3.7 Feature importance and model interpretability analysis

A feature importance analysis of the CatBoost machine learning model is shown in **Supplementary Figure S6A**. The SHAP summary plot (**Supplementary Figure S8B**) provides a visual representation of the predictive contributions of individual variables in the model. Similarly, a dual-coordinate line plot (**Supplementary Figure S8C**) revealed how each feature influenced the model’s predictive outcomes, with each feature’s SHAP value displayed along a line segment.

Moreover, the age distribution of mCRC patients exhibited certain characteristics; the clinical manifestations and prognoses may be closely associated with age (**Supplementary Figure S8D**). Younger patients often possess stronger physiological reserves and recovery capabilities, and studies have suggested that younger patients may exhibit more aggressive disease progression. In contrast, older patients may be affected by their treatment choices and the prognosis may be influenced by comorbidities or poor overall health.

## 4 Discussion

Our findings support the initial hypothesis by evaluating the predictive capabilities of a model for selecting candidates for primary tumor surgery in mCRC. The positive outcomes highlight the efficacy of PTR for mCRC. The traditional logistic model exhibited superior performance compared with machine learning models, providing clinicians with a reliable tool to estimate the potential benefits of surgery for patients.

Stage IV CRC patients are typically managed with systemic, palliative, or end-of-life care, and local treatments, including PTR, are avoided (5). However, considerable heterogeneity among mCRC patients, including variations in age, histological subtypes, and chemotherapy protocols, can affect prognosis (18). Some studies have questioned the benefits of PTR for mCRC patients. A Japanese randomized controlled trial demonstrated no notable difference in overall survival between the surgery and non-surgery cohorts (median OS of 25.9 *vs*. 26.4 months, P<0.05), suggesting that PTR might not improve survival in CRC patients (19). However, Lam-Boer et al. (20) and Doah et al. (21)used PSM to reduce selection bias and reported benefits of PTR for advanced CRC. Furthermore, studies indicate that 7-22% of patients without an initial PTR require emergency surgeries (22–24). Wang et al. observed that PTR improved quality-of-life as well as reduced the risk of severe problems including bleeding and perforation (25).

Consistent with previous reports, our study demonstrated that PTR was associated with improved survival in patients with stage IV mCRC, as indicated by the Cox regression analysis (20, 26, 27). However, after applying the competing risk model, PTR did not significantly improve CSS when non-cancer-related death was considered as a competing event. Notably, some patients who underwent surgery did not reach a median CSS of 12 months, suggesting that surgical intervention may not benefit all individuals. These findings highlight the limitations of current surgical recommendations and underscore the need for more selective patient stratification. For patients unlikely to benefit from PTR, non-surgical management or palliative care strategies should be considered as alternative approaches.

Surgical intervention may improve survival due to selection bias; however, this is not the sole factor. Herein, chemotherapy and age were key predictors of surgical benefits. Previous studies have observed that stage IV CRC patients receiving both PTR and chemotherapy had a median OS of 23 months than those receiving only chemotherapy (13 months), sorely surgery (6 months), or without intervention (2 months) (28). In some studies, the age varied from 60 to 75 years, with no significant differences across treatment groups. Our data (**Supplementary Figure S9A**) suggests that PTR with systemic chemotherapy provides greater benefits than PTR alone. Additionally, younger patients benefited more from PTR (**Supplementary Figure S9B**), underscoring the importance of individual characteristics in surgical decision-making. This finding suggests that healthier patients with longer life expectancies are more likely to choose aggressive treatments, including surgery.

The 2018 TNM staging system for mCRC was updated to classify metastases as M1a, M1b, and M1c. Some M1c cases were not distinguishable in the SEER database; therefore, staging was redefined using the 7th edition of the AJCC system to ensure sample representativeness. This staging strategy may affect prognostic interpretation following PTR in mCRC patients. Research indicates that complete removal of the primary neoplasm and metastatic masses through PTR can prolong survival and quality-of-life in M1b cases. However, M1c cases with more widespread disease distribution may experience limited treatment effectiveness and survival (29, 30). Combining M1b and M1c stages may complicate the assessment of the metastatic burden on prognosis. Accurate determination of the M1c stage in clinical practice requires expensive imaging studies. Despite our model not differentiating between M1b and M1c, it still exhibited strong predictive capabilities.

In the surgical benefit prediction model developed in this study, patients with stage III/IV disease and T4 tumors exhibited a lower probability of deriving benefit from PTR, as reflected by lower odds ratios. This finding aligns with the intrinsic relationship between tumor biology and surgical feasibility. T4 tumors are typically characterized by aggressive local invasion and a higher likelihood of involving adjacent organs or structures, which complicates surgical resection and reduces the likelihood of achieving curative outcomes. Similarly, stage III/IV disease indicates more extensive regional or distant metastasis, suggesting a higher degree of systemic tumor progression. In such patients, even if PTR is technically feasible, the overall survival benefit may be limited and could be accompanied by an increased risk of postoperative complications. Therefore, the decision for surgical intervention should not rely solely on anatomical resectability but must also incorporate a thorough evaluation of tumor biology and progression patterns. The findings from our prediction model underscore this principle, highlighting the central role of tumor biology in guiding surgical decision-making.

Although this study is based on the SEER database, which provides a large sample size and high data completeness for model development, several inherent structural limitations may affect the interpretation of our results. First, the SEER database does not systematically record the specific sites of distant metastases (e.g., liver or lung) or the number of metastatic lesions. This limitation prevents us from distinguishing oligometastatic disease from widely metastatic cases. In clinical practice, such distinctions are critical for surgical decision-making, particularly when assessing the suitability of PTR in patients with mCRC. Second, patient performance status data, such as Eastern Cooperative Oncology Group (ECOG) scores or Karnofsky Performance Status (KPS), are not available in SEER. As a result, we could not directly evaluate physical condition or surgical tolerance in our model, which may introduce risk stratification bias. Although we used propensity score matching to balance available covariates such as age and comorbidities, the lack of functional status indicators remains a limitation to the model’s generalizability. In addition, SEER does not capture information on disease recurrence or postoperative complications. This prevents a comprehensive assessment of long-term recurrence risks and non-cancer-related postoperative mortality, potentially leading to an underestimation of long-term outcomes. Although SEER ensures high survival data accuracy through linkage to sources such as the National Death Index, follow-up time may still vary across patients. To address this, we used the reverse Kaplan-Meier method to estimate the median follow-up time, which was 65 months. This indicates an overall adequate follow-up period. However, right-censoring bias may still occur in long-term survivors due to unobserved late events. These limitations—particularly the absence of data on metastasis burden and performance status—may reduce the accuracy and clinical applicability of the predictive model in guiding PTR decisions for stage IV disease. The lack of treatment-specific variables also limits our understanding of how PTR and systemic therapy interact to affect outcomes. Future studies should consider integrating multicenter clinical datasets or real-world electronic health records (EHRs). Such data sources can provide a more comprehensive set of preoperative variables, including metastatic load, functional status, and postoperative complications. This would help improve the accuracy, clinical relevance, and decision-support capability of predictive models for mCRC.

## 5 Conclusion

We present an approach to identify suitable candidates for surgical intervention among stage IV CRC patients. Notwithstanding the widespread adoption of machine learning, traditional logistic regression models still demonstrate competitive predictive capabilities. Our findings revealed that PTR can positively impact mCRC patients. However, this is limited to specific patient groups, and the extent of the benefits is influenced by the features of primary tumor. Specifically, younger patients and those with cystic/mucinous and serous tumors, Grade II, T2 stage, M1a stage, undergoing chemotherapy, and surgery at distant sites are likely to benefit more from PTR.

## Data Availability

Publicly available datasets were analyzed in this study. This data can be found here: https://seer.cancer.gov/.

## Supplementary material

Supplementary Figure 1 Flowchart of research population selection and prediction model construction.

Supplementary Figure 2 Evaluation of Dataset Balance and Variable Matching Before and After PSM. **(A)** The improvement in dataset balance after PSM. The y-axis quantifies the SMD, with values closer to zero after PSM demonstrating better balance. **(B)** Differences in variables before and after matching. Abbreviation: PSM, propensity score matching; SMD, standard mean difference.

Supplementary Figure 3 Kaplan-Meier plot of mCRC patients according to treatment. Abbreviation: mCRC, metastatic colorectal cancer; SEER, Surveillance, Epidemiology and End Results; PSM, propensity score matching; CSS, cancer specific survival; OS, overall survival; HR, hazard ratio.

Supplementary Figure 4 Cumulative incidence functions for cancer-specific and non-cancer-specific death in patients with mCRC, stratified by surgical treatment. The figure displays cumulative incidence functions (CIFs) derived from the Fine-Gray competing risk model, comparing patients with mCRC who underwent PTR versus those who did not. Cancer-specific death (event type 1) and non-cancer-related death (event type 2) are shown separately for the PTR group (group 1) and the non-PTR group (group 0).Lines labeled “0 1” and “1 1” represent cancer-specific mortality in the non-PTR and PTR groups, respectively. Lines labeled “0 2” and “1 2” represent non-cancer mortality in the respective groups. The curves indicate similar cancer-specific mortality across both groups, with slightly higher non-cancer mortality observed in the PTR group.

Supplementary Figure 5 Clinical Impact and ROC Curves for Risk Prediction. Training **(A)** and test **(B)** set ROC curves, each based on 500 bootstrap samples to assess the stability and robustness of the model's performance. **(C)** illustrates the variability and confidence intervals of the model in the training set, while **(D)** depicts the same for the test set. Clinical impact curves plot the percentage of individuals classified as high risk and those who actually experience the event across various high-risk thresholds in the training **(E)** and test **(F)** sets. ROC: receiver operating matching.

Supplementary Figure 6 Assessment of Model Validity Using ROC and DCA Curves for Training and Test Sets. This analysis provides a comparative view of how each variable contributes to the prediction accuracy and clinical decision-making.

Supplementary Figure 7 Assessment of Validity Based on Nomoscore. The effectiveness of the Nomoscore in distinguishing between the low score and high score in the training **(A)** and test **(B)** sets. **(C)** The odds ratios increase with higher Nomoscore quartiles, indicating a greater likelihood of the outcome as the score increases. all significantly higher than the reference group, demonstrating a strong positive association between the Nomoscore and the observed outcome.

Supplementary Figure 8 SHAP Analysis Visualizations for a CatBoost Machine Learning Model. **(A)** This bar chart ranks the features by their importance based on the average magnitude of SHAP values. **(B)** Showing the spectrum from negative to positive contributions. **(C)** SHAP HeatWave Plot shows the SHAP values across all data points over time, illustrating the influence of features Age, Grade, and Histologic type on model predictions. **(D)** This scatter plot maps the SHAP values against Age, demonstrating how the influence of age varies across different SHAP values. Abbreviation: CatBoost, Categorical Boosting; SHAP, shapley additive explanations.

Supplementary Figure 9 Distribution of Age and Chemotherapy by Benefit Status. The age is lower in the surgery benefit group **(A)**, and patients who receive chemotherapy are more likely to benefit from surgery **(B)**.
