# Supplementary material for: A predictive model to identify optimal candidates for surgery among patients with metastatic colorectal cancer
Source: Front Oncol. 2025 Jun 5;15:1573431. doi: 10.3389/fonc.2025.1573431 (PMC12176591; doi:10.3389/fonc.2025.1573431)
Supplement: Supplementary Figure 1 — Flowchart of research population selection and prediction model construction. [file DataSheet1.pdf]

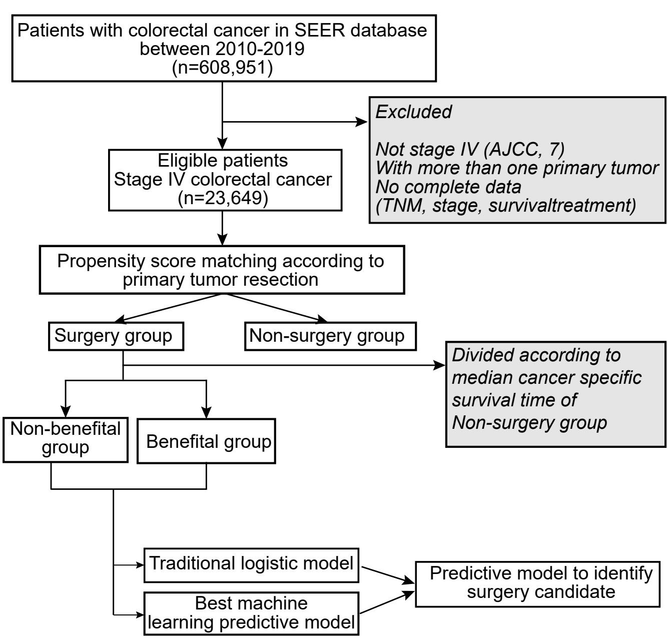
Patients with colorectal cancer in SEER database between 2010-2019 (n=608,951)
Excluded
Not stage IV (AJCC, 7)
With more than one primary tumor
No complete data
(TNM, stage, survivaltreatment)
Eligible patients
Stage IV colorectal cancer
(n=23,649)
Propensity score matching according to primary tumor resection
Surgery group
Non-surgery group
Divided according to median cancer specific survival time of Non-surgery group
Non-benefital group
Benefital group
Traditional logistic model
Best machine learning predictive model
Predictive model to identify surgery candidate
