# Supplementary material for: A predictive model to identify optimal candidates for surgery among patients with metastatic colorectal cancer
Source: Front Oncol. 2025 Jun 5;15:1573431. doi: 10.3389/fonc.2025.1573431 (PMC12176591; doi:10.3389/fonc.2025.1573431)
Supplement: Supplementary file 10 [file DataSheet10.zip › Supplementary Table 1.docx]

| **Supplementary Table S1 Comparison of differences in cancer specific survival time after PSM.** | | | | | |
| --- | --- | --- | --- | --- | --- |
|  |  | Group | |  |  |
| Variable Names | Total (n=4817) | Surgery to primary site (n=2404),n% | Non-surgery to primary site (n=2413),n% | P value | SMD |
| Survival months | 16 (6-32) | 22 (9-41) | 12 (4-24) | <0.01 | 0.53 |

PSM, propensity score matching
