# Supplementary material for: A predictive model to identify optimal candidates for surgery among patients with metastatic colorectal cancer
Source: Front Oncol. 2025 Jun 5;15:1573431. doi: 10.3389/fonc.2025.1573431 (PMC12176591; doi:10.3389/fonc.2025.1573431)
Supplement: Supplementary file 10 [file DataSheet10.zip › Supplementary Table 2.docx]

| **Supplementary Table S2 Comparison of baseline performance on Training and Testing sets.** | | | | |
| --- | --- | --- | --- | --- |
|  |  | Group | |  |
| Variable Names | Overall(n=2404),n% | Train(n=1442),n% | Test(n=962),n% | P value |
| **Age** | 61.00(53.00,71.00) | 61.00(53.00,71.00) | 62.00(53.00,71.00) | 0.491 |
| **Gender** |  |  |  | 0.657 |
| Male | 1380(57.40) | 822(57.00) | 558(58.00) |  |
| Female | 1024(42.60) | 620(43.00) | 404(42.00) |  |
| **Marriage** |  |  |  | 0.670 |
| Married | 1266(52.66) | 765(53.05) | 501(52.08) |  |
| Never married/other | 1138(47.34) | 677(46.95) | 461(47.92) |  |
| **Race** |  |  |  | 0.978 |
| White | 1842(76.62) | 1107(76.77) | 735(76.40) |  |
| Black | 315(13.10) | 188(13.04) | 127(13.20) |  |
| Other | 247(10.27) | 147(10.19) | 100(10.40) |  |
| **Site** |  |  |  | 0.169 |
| Right-hemicolon | 609(25.33) | 347(24.06) | 262(27.23) |  |
| Left-hemicolon | 542(22.55) | 324(22.47) | 218(22.66) |  |
| Rectum | 1253(52.12) | 771(53.47) | 482(50.10) |  |
| **Histologic** |  |  |  | 0.805 |
| Adenomas and adenocarcinomas | 2206(91.76) | 1319(91.47) | 887(92.20) |  |
| Cystic, mucinous and serous neoplasms | 149(6.20) | 93(6.45) | 56(5.82) |  |
| Other | 49(2.04) | 30(2.08) | 19(1.98) |  |
| **Grade** |  |  |  | 0.913 |
| Ⅰ | 170(7.07) | 98(6.80) | 72(7.48) |  |
| Ⅱ | 1688(70.22) | 1015(70.39) | 673(69.96) |  |
| Ⅲ | 491(20.42) | 297(20.60) | 194(20.17) |  |
| Ⅳ | 55(2.29) | 32(2.22) | 23(2.39) |  |
| **T** |  |  |  | 0.608 |
| T1 | 274(11.40) | 157(10.89) | 117(12.16) |  |
| T2 | 108(4.49) | 63(4.37) | 45(4.68) |  |
| T3 | 1187(49.38) | 731(50.69) | 456(47.40) |  |
| T4a | 142(5.91) | 83(5.76) | 59(6.13) |  |
| T4b | 693(28.83) | 408(28.29) | 285(29.63) |  |
| **N** |  |  |  | 0.228 |
| N0 | 1049(43.64) | 619(42.93) | 430(44.70) |  |
| N1 | 1134(47.17) | 683(47.36) | 451(46.88) |  |
| N1c | 45(1.87) | 30(2.08) | 15(1.56) |  |
| N2a | 79(3.29) | 43(2.98) | 36(3.74) |  |
| N2b | 97(4.03) | 67(4.65) | 30(3.12) |  |
| **M** |  |  |  | 0.100 |
| M1a | 1270(52.83) | 782(54.23) | 488(50.73) |  |
| M1b | 1134(47.17) | 660(45.77) | 474(49.27) |  |
| **Radiation** |  |  |  | 0.833 |
| Yes | 609(25.33) | 368(25.52) | 241(25.05) |  |
| None/unknown | 1795(74.67) | 1074(74.48) | 721(74.95) |  |
| **Chemotherapy** |  |  |  | 0.477 |
| Yes | 1804(75.04) | 1090(75.59) | 714(74.22) |  |
| None/unknown | 600(24.96) | 352(24.41) | 248(25.78) |  |
| **Surgery other sites** |  |  |  | 0.829 |
| Yes | 210(8.74) | 124(8.60) | 86(8.94) |  |
| None/unknown | 2194(91.26) | 1318(91.40) | 876(91.06) |  |
| To split the surgery group patients into training and testing sets using a 6:4 ratio | | | | |
