# Supplementary material for: A predictive model to identify optimal candidates for surgery among patients with metastatic colorectal cancer
Source: Front Oncol. 2025 Jun 5;15:1573431. doi: 10.3389/fonc.2025.1573431 (PMC12176591; doi:10.3389/fonc.2025.1573431)
Supplement: Supplementary file 10 [file DataSheet10.zip › Supplementary Table 3.docx]

| **Supplementary Table S3 Inclusion of Variables for Brouta Feature Selection.** | | | | | | |
| --- | --- | --- | --- | --- | --- | --- |
| Variable Names | meanImp | medianImp | minImp | maxImp | normHits | decision |
| Age | 11.9168971 | 11.5716814 | 7.42822578 | 17.5242211 | 1 | Confirmed |
| Marriage | -0.481377 | 0.1440858 | -3.48918651 | 0.9486625 | 0 | Rejected |
| Race | 2.2728273 | 2.1014051 | -1.05381158 | 7.4766343 | 0.41414141 | Tentative |
| Site | 4.8750207 | 4.7622498 | 1.29731275 | 11.3868028 | 0.87878788 | Confirmed |
| Histologic | 5.0849529 | 4.7922132 | 2.40552846 | 8.6943223 | 0.86868687 | Confirmed |
| Grade | 2.1778776 | 2.2863619 | -1.73177054 | 5.8525094 | 0.37373737 | Tentative |
| T | 6.9175442 | 6.8426037 | 2.20290679 | 11.5032267 | 0.94949495 | Confirmed |
| N | 0.4804071 | 0.575868 | -2.01494928 | 3.8822274 | 0.01010101 | Rejected |
| M | 6.6722256 | 6.6307513 | 2.30849631 | 12.3005383 | 1 | Confirmed |
| Chemotherapy | 48.5350137 | 48.7455711 | 42.65505564 | 53.9364167 | 1 | Confirmed |
| Surgery other sites | 3.7088142 | 3.7195455 | -0.04267351 | 7.4415793 | 0.60606061 | Tentative |
| It is based on the random forest classification method and works by creating a shadow feature for each original feature in the dataset. | | | | | | |

Boruta is a feature ranking and selection algorithm aimed at identifying all relevant features in a dataset for predictive modeling. It is based on the random forest classification method and works by creating a shadow feature for each original feature in the dataset.

**Confirmed**: These features are confirmed by the Boruta algorithm as important, meaning their importance significantly exceeds that of the corresponding shadow features. This indicates that these features play a significant role in the predictive model and should be included in the final model.

**Rejected**: These features are marked as unimportant by the Boruta algorithm, as their importance is lower than or does not significantly exceed the importance of the shadow features. This suggests that these features do not contribute significantly to the model's predictive ability and are typically excluded from the final model.

**Tentative**: The importance status of these features remains undecided. In the iterative process of the Boruta algorithm, if a feature is neither confirmed as important nor fully rejected, its status is marked as tentative. Further iterations or adjustments are needed to determine the importance of these features.
