# Supplementary material for: A predictive model to identify optimal candidates for surgery among patients with metastatic colorectal cancer
Source: Front Oncol. 2025 Jun 5;15:1573431. doi: 10.3389/fonc.2025.1573431 (PMC12176591; doi:10.3389/fonc.2025.1573431)
Supplement: Supplementary file 10 [file DataSheet10.zip › Supplementary Table 4.docx]

| **Supplementary Table S4 Performance metrics of the machine-learning models and traditional logistic model in the test set queue.** | | | | |
| --- | --- | --- | --- | --- |
| ModelName | Recall | Accuracy | F1-Score | MCC |
| GBDT | 0.782 | 0.661 | 0.755 | 0.211 |
| CatBoost | 0.885 | 0.747 | 0.824 | 0.398 |
| Naïve_Bayes | 0.878 | 0.709 | 0.801 | 0.291 |
| RandomForest | 0.902 | 0.740 | 0.822 | 0.373 |
| Logistic Classifier | 0.872 | 0.732 | 0.813 | 0.360 |
| SVC | 1.000 | 0.666 | 0.800 | NA |
| AdaBoost | 0.733 | 0.651 | 0.737 | 0.218 |
| LGBM | 0.858 | 0.735 | 0.812 | 0.374 |
| XGBoost | 0.852 | 0.729 | 0.807 | 0.360 |
| Traditional Logistic | 0.777 | 0.721 | 0.788 | NA |

AdaBoost, Adaptive Boosting; CatBoost, Categorical Boosting; GBDT, Gradient Boosting Trees; LightGBM, Light Gradient Boosting Machine; SVM, Support vector machine; XGBoost, eXtreme Gradient Boosting.
