# Supplementary material for: A predictive model to identify optimal candidates for surgery among patients with metastatic colorectal cancer
Source: Front Oncol. 2025 Jun 5;15:1573431. doi: 10.3389/fonc.2025.1573431 (PMC12176591; doi:10.3389/fonc.2025.1573431)
Supplement: Supplementary file 10 [file DataSheet10.zip › Supplementary Table 5.docx]

| **Supplementary Table S5 Performance Metrics of Nomogram-Based Probability Predictions Across Different Datasets.** | | | | | | | | | | | | | | |
| --- | --- | --- | --- | --- | --- | --- | --- | --- | --- | --- | --- | --- | --- | --- |
| Datatset | Cutoff | AUC | P | ACC | SEN | SPE | PLR | NLR | PPV | NPV | PPA | NPA | TPA | KAPPA |
| Trianset_raw | 0.655 | 0.727 | 0 | 0.723 | 0.8 | 0.564 | 1.836 | 0.355 | 0.79 | 0.579 | 0.8 | 0.564 | 0.723 | 0.367 |
| Trianset_binary | 0.655 | 0.682 | 0 | 0.723 | 0.8 | 0.564 | 1.836 | 0.355 | 0.79 | 0.579 | 0.8 | 0.564 | 0.723 | 0.367 |
| Validationset_raw | 0.676 | 0.741 | 0 | 0.725 | 0.76 | 0.654 | 2.197 | 0.367 | 0.814 | 0.577 | 0.76 | 0.654 | 0.725 | 0.401 |
| Validationset_binary | 0.655 | 0.692 | 0 | 0.72 | 0.777 | 0.607 | 1.979 | 0.367 | 0.798 | 0.577 | 0.777 | 0.607 | 0.72 | 0.379 |
|  | | | | | | | | | | | | | | |

AUC,Area Under the Curve - Measures the ability of the model to discriminate between positive and negative classes;P,P-value - Indicates the statistical significance of the observed effect;ACC,Accuracy - Proportion of true results (both true positives and true negatives) among the total number of cases examined;SEN, Sensitivity - The probability that a test results positive when the condition is present (also known as true positive rate or recall);SPE, Specificity - The probability that a test results negative when the condition is absent (true negative rate);PLR,Positive Likelihood Ratio - Ratio of the probability of a positive test result given the presence of the condition to the probability of a positive test result given the absence of the condition;NLR, Negative Likelihood Ratio - Ratio of the probability of a negative test result given the presence of the condition to the probability of a negative test result given the absence of the condition;PPV, Positive Predictive Value - Probability that the condition is present when the test is positive;NPV,Negative Predictive Value - Probability that the condition is absent when the test is negative.;PPA,Positive Percent Agreement - Agreement percentage of the positive cases between two different testing methods;NPA, Negative Percent Agreement - Agreement percentage of the negative cases between two different testing methods;TPA, Total Percent Agreement - Overall agreement percentage between two different testing methods;KAPPA, Kappa Statistic - A statistical measure that compares an Observed Accuracy with an Expected Accuracy (random chance).
