# Supplementary material for: A predictive model to identify optimal candidates for surgery among patients with metastatic colorectal cancer
Source: Front Oncol. 2025 Jun 5;15:1573431. doi: 10.3389/fonc.2025.1573431 (PMC12176591; doi:10.3389/fonc.2025.1573431)
Supplement: Supplementary file 11 [file SupplementaryFile1.docx]

Supplementary Figure Legends

**Supplementary Figure S1 Flowchart of research population selection and prediction model construction.**

**Supplementary Figure** S**2 Evaluation of Dataset Balance and Variable Matching Before and After PSM.** (A) The improvement in dataset balance after PSM. The y-axis quantifies the SMD, with values closer to zero after PSM demonstrating better balance. (B) Differences in variables before and after matching. Abbreviation: PSM, propensity score matching; SMD, standard mean difference.

**Supplementary Figure** S**3 Kaplan-Meier plot of mCRC patients according to treatment.** Abbreviation: mCRC, metastatic colorectal cancer; SEER, Surveillance, Epidemiology and End Results; PSM, propensity score matching; CSS, cancer specific survival; OS, overall survival; HR, hazard ratio.

**Supplementary Figure 4 Cumulative incidence functions for cancer-specific and non-cancer-specific death in patients with mCRC, stratified by surgical treatment.** The figure displays cumulative incidence functions (CIFs) derived from the Fine-Gray competing risk model, comparing patients with mCRC who underwent PTR versus those who did not. Cancer-specific death (event type 1) and non-cancer-related death (event type 2) are shown separately for the PTR group (group 1) and the non-PTR group (group 0).Lines labeled “0 1” and “1 1” represent cancer-specific mortality in the non-PTR and PTR groups, respectively. Lines labeled “0 2” and “1 2” represent non-cancer mortality in the respective groups. The curves indicate similar cancer-specific mortality across both groups, with slightly higher non-cancer mortality observed in the PTR group.

**Supplementary Figure S5 Clinical Impact and ROC Curves for Risk Prediction.** Training(A) and test(B) set ROC curves, each based on 500 bootstrap samples to assess the stability and robustness of the model's performance. (C) illustrates the variability and confidence intervals of the model in the training set, while (D) depicts the same for the test set. Clinical impact curves plot the percentage of individuals classified as high risk and those who actually experience the event across various high-risk thresholds in the training(E) and test(F) sets. ROC: receiver operating matching.

**Supplementary Figure S6 Assessment of Model Validity Using ROC and DCA Curves for Training and Test Sets.** This analysis provides a comparative view of how each variable contributes to the prediction accuracy and clinical decision-making.

**Supplementary Figure S7 Assessment of Validity Based on Nomoscore.** The effectiveness of the Nomoscore in distinguishing between the low score and high score in the training(A) and test(B) sets. (C) The odds ratios increase with higher Nomoscore quartiles, indicating a greater likelihood of the outcome as the score increases. all significantly higher than the reference group, demonstrating a strong positive association between the Nomoscore and the observed outcome.

**Supplementary Figure S8 SHAP Analysis Visualizations for a CatBoost Machine Learning Model.** (A) This bar chart ranks the features by their importance based on the average magnitude of SHAP values. (B)Showing the spectrum from negative to positive contributions. (C)SHAP HeatWave Plot shows the SHAP values across all data points over time, illustrating the influence of features Age, Grade, and Histologic type on model predictions. (D)This scatter plot maps the SHAP values against Age, demonstrating how the influence of age varies across different SHAP values. Abbreviation: CatBoost, Categorical Boosting; SHAP, shapley additive explanations.

**Supplementary Figure S9 Distribution of Age and Chemotherapy by Benefit Status.** The age is lower in the surgery benefit group(A), and patients who receive chemotherapy are more likely to benefit from surgery(B).
