# Supplementary material for: A predictive model to identify optimal candidates for surgery among patients with metastatic colorectal cancer
Source: Front Oncol. 2025 Jun 5;15:1573431. doi: 10.3389/fonc.2025.1573431 (PMC12176591; doi:10.3389/fonc.2025.1573431)
Supplement: Supplementary Figure 3 — Kaplan-Meier plot of mCRC patients according to treatment. Abbreviation: mCRC, metastatic colorectal cancer; SEER, Surveillance, Epidemiology and End Results; PSM, propensity score matching; CSS, cancer specific survival; OS, overall survival; HR, hazard ratio. [file DataSheet3.pdf]

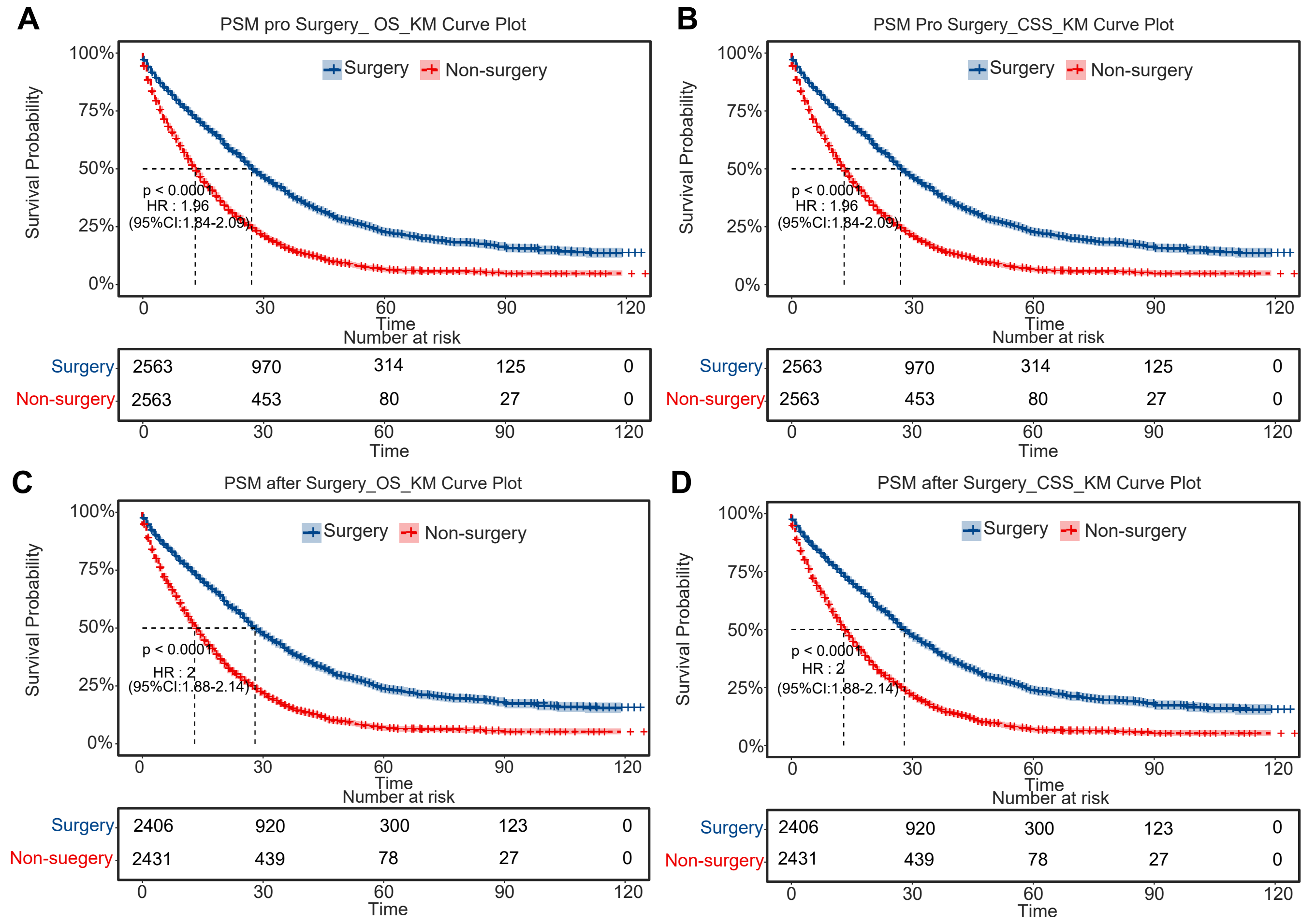
A
PSM pro Surgery_ OS_KM Curve Plot
Survival Probability
100%
75%
50%
25%
0%
Surgery
Non-surgery
p < 0.0001
HR : 1.96
(95%CI:1.84-2.09)
0 30 60 90 120
Time
Number at risk
Surgery 2563 970 314 125 0
Non-surgery 2563 453 80 27 0
0 30 60 90 120
Time
B
PSM Pro Surgery_CSS_KM Curve Plot
Survival Probability
100%
75%
50%
25%
0%
Surgery
Non-surgery
p < 0.0001
HR : 1.96
(95%CI:1.84-2.09)
0 30 60 90 120
Time
Number at risk
Surgery 2563 970 314 125 0
Non-surgery 2563 453 80 27 0
0 30 60 90 120
Time
C
PSM after Surgery_OS_KM Curve Plot
Survival Probability
100%
75%
50%
25%
0%
Surgery
Non-surgery
p < 0.0001
HR : 2
(95%CI:1.88-2.14)
0 30 60 90 120
Time
Number at risk
Surgery 2406 920 300 123 0
Non-suegery 2431 439 78 27 0
0 30 60 90 120
Time
D
PSM after Surgery_CSS_KM Curve Plot
Survival Probability
100%
75%
50%
25%
0%
Surgery
Non-surgery
p < 0.0001
HR : 2
(95%CI:1.88-2.14)
0 30 60 90 120
Time
Number at risk
Surgery 2406 920 300 123 0
Non-surgery 2431 439 78 27 0
0 30 60 90 120
Time
